# Associations between sleep characteristics and weight gain in an older population: results of the Heinz Nixdorf Recall Study

**DOI:** 10.1038/nutd.2016.32

**Published:** 2016-08-15

**Authors:** B Kowall, A-T Lehnich, R Erbel, S Moebus, K-H Jöckel, A Stang

**Affiliations:** 1 Center of Clinical Epidemiology, Institute for Medical Informatics, Biometry and Epidemiology (IMIBE), Medical Faculty, University Duisburg-Essen, Essen, Germany; 2 Institute for Medical Informatics, Biometry and Epidemiology, University Hospital Essen, University Duisburg-Essen, Essen, Germany; 3 Center for Urban Epidemiology, Institute for Medical Informatics, Biometry, and Epidemiology, University Hospital Essen, Essen, Germany; 4 Department of Epidemiology, School of Public Health, Boston University, Boston, MA, USA

## Abstract

**Background/Objectives:**

Sleep duration influences weight change in children and young adults, but there is less evidence in middle-aged, and, in particular, older adults. We assessed associations between sleep duration, daytime napping and sleep disturbances, respectively, with change of weight and waist circumference in older subjects. Contrary to previous studies, we also used two points in time to assess sleep characteristics.

**Methods:**

We used data from the population-based Heinz Nixdorf Recall study, a cohort study in Germany with a baseline and two follow-up visits (age 45–74 years, median follow-up 5.1 years for first, 5.2 years for second follow-up visit). In adjusted linear regression models (*N*=3751), we estimated weight change between baseline and first follow-up visit in relation to various self-reported sleep characteristics measured at baseline. Furthermore, we estimated change of weight and waist circumference, respectively, between first and second follow-up visit in relation to patterns of sleep characteristics measured at baseline and at the first follow-up visit (*N*=2837).

**Results:**

In all analyses, short and long sleep duration, sleep disturbances, and regular daytime napping were associated with <1 kg of weight gain and <1 cm of gain in waist circumference over 5 years compared with the respective reference categories. For example, compared with 7–<8 h night sleep, short night sleep (⩽5 h at baseline) was associated with 0.5 kg of weight gain (95% confidence interval: −0.1; 1.1 kg).

**Conclusions:**

Our study gave no evidence that sleep characteristics were associated with clinically relevant weight gain in the older population.

## Introduction

In 2012, Magee and Hale published a systematic review on prospective studies of the association between nocturnal sleep duration and weight gain.^1^ They concluded that short sleep is associated with weight gain in children but that results from 13 adult studies were inconclusive. After the Magee and Hale review, more longitudinal studies on sleep duration and weight gain in adults were published.^2, 3, 4, 5, 6, 7^ In three recent studies from Japan, either no association,^2^ an association with a very small BMI gain (<0.1 kg m^−^^2^ over 3 years),^3^ or an association between short nocturnal sleep and weight gain only in shift workers^4^ were reported. In two other recent studies from the USA, one showed a small weight gain (<0.7 kg over 7.5 years) in short sleepers (<5 h),^5^ and in the other, an association between short sleep duration and incidence of obesity was reported for subjective, but not for objective sleep duration.^6^ In 2014, Wu *et al.*^8^ pooled odds ratios for sleep duration and incident obesity in adults, indicating an increased obesity odds for short, but not for long nocturnal sleep. There are some hints that sleep duration has a larger impact on weight change in younger adults compared with the middle-aged and older adults,^9, 10^ but there is still a lack of research on this question.

Important methodical problems have been identified in studies on sleep duration and weight gain.^11, 12^ In the present study, we addressed some critical points. First, sleep characteristics only reported at one point in time may be transient and be due to temporary factors, and, therefore, we hypothesized that stronger associations with weight change may be identified for sleep characteristics reported more than once. Contrary to previous studies, we also used patterns of sleep characteristics from two points in time to predict weight change in the present study. Second, in many studies, only nocturnal sleep was taken into account. In this study, we also considered daytime napping and total sleep time. Third, in previous studies, there is considerable heterogeneity in the choice of reference categories of sleep duration. In this study, we assessed whether variations in the reference category have an impact on associations between sleep duration and weight change.

For the present analyses, we used data of a population-based cohort study with older participants. Subjects were invited for one baseline and two follow-up visits, so that three study points were available for each participant. Our aim was to assess associations between nocturnal sleep duration, daytime napping, total sleep duration, and sleep disturbances, respectively, and subsequent weight change (and subsequent change in waist circumference, respectively).

## Materials and methods

### Study population

The Heinz Nixdorf Recall study is a population-based prospective cohort study conducted in three large adjacent cities (Bochum, Essen, Mülheim) in the Ruhr-region in North-Rhine-Westphalia in Germany. The study rationale and design have been described in detail elsewhere.^13^ In short, the cohort comprises a total of 4814 subjects (49.8% men, aged 45–75 years). The baseline visits were performed between 2000 and 2003. The median follow-up was 5.1 years for the 5-year follow-up visits between 2005 and 2008, and 5.2 years for the 5-year follow-up visits between 2011 and 2015. Data assessment at baseline and at follow-up visits included a self-administered questionnaire, face-to-face interviews, and a physical examination including among others anthropometric measurements and comprehensive laboratory tests. The study was approved by the relevant institutional ethics committees. All participants gave their written informed consent.

In the following, T0 means the time of the baseline assessment (2000–2003), T1 means the time of the second visit (2005–2008), and T2 means the time of the third visit (2011–2015).

### Assessment of sleep characteristics

Self-reported sleep characteristics were assessed in an identical way at T0 and T1. The interview included one open question on average nocturnal sleep duration (‘For how many hours do you sleep on average at night?' Subjects were asked to state hours and minutes). Frequency of daytime napping (‘How often do you normally take a nap?') was assessed as never, less than once a week, 1–4 times per week, 5–6 times per week, and daily. All subjects except for never nappers were asked a question on nap duration (‘For how many hours do you normally take a nap?' Subjects were asked to state hours and minutes). Mean duration of daytime napping was calculated as reported duration of daytime napping times its relative frequency. Total sleep duration was the sum of nocturnal sleep duration and mean duration of daytime napping.

Questions on nocturnal sleep disturbances during the last four weeks covered difficulties falling asleep, difficulties maintaining sleep, and early morning arousal (‘How often did it occur to you in the last four weeks: that you had difficulties falling asleep/that you woke up at night/that you woke up earlier as usual?'). Frequency of nocturnal sleep disturbances was assessed as never, sometimes (⩽1 times per week), frequently (⩾2 times per week), and nearly every night. Sleep disturbances occurring nearly every night were defined as regular.

### Assessment of outcome

Data on weight were collected with measuring systems of the company ‘seca' (seca GmbH & Co. kg, Hamburg, Germany). According to the manufacturer, the precision of the measurement system SECA 709 was 100 g. Waist circumference was measured to the nearest 0.1 cm using a flexible, inelastic tape. It was measured at the narrowest part between the lowest rib and the highest point of the iliac crest. All participants wore light underwear.

### Covariates

Covariates for model adjustment were selected according to known predictors of weight gain, and factors that have an influence on sleep duration.^14, 15, 16^ In the examinations at T0 and at T1, interviews were conducted to gather information on smoking status, alcohol, diet, physical activity, school education and marital status. Smoking was grouped into three categories (current, former, never smoker). Average pure ethanol intake in g day^−1^ was estimated from frequencies of drinking beer, wine, sparkling wine and spirits.^17^ At T0, information on kind and duration of exercise performed in the preceding month was used to estimate metabolic equivalents per week.^18^ At T1, amount of sport per week was assessed as no/<1 h/1–2 h/more than 2 h in a typical week. An index indicating accordance with dietary guidelines of the German Society of Nutrition was derived from a food frequency questionnaire with information on the consumption frequency of 13 food items.^19, 20^ This index was coded as low (<12), medium (12–14) and high (⩾15). School education was coded as low (<10 years), medium (10 years) and high (>10 years). Subjective health status was categorized as very good, good, satisfactory, poor or very poor.

To assess stress at T0, we used a 13-item scale on problems with housework, partners, children, acquaintances and money. Participants were asked, whether they had such problems, and, if so, to what degree these problems bothered them. The scale and calculation of a stress score are described in detail in Supplementary Table 6.

### Statistical analyses

To estimate the association between weight change between T0 and T1 and sleep characteristics at T0, we used a dataset with complete data on sleep duration and covariates at T0, and weight at T0 and T1, leaving a total of 3751 participants. We fitted linear regression models with weight change between T0 and T1 as the outcome and the following sleep characteristics at T0 as the exposure variable:

- Duration of night sleep using different categories:
  ○ (<6 h; >8 h; 6–8 h (reference))
  ○ (<5 h; 5–5.9 h; >8 h; 6–8 h (reference))
  ○ (⩽5 h; 5.1–6.9 h; ⩾8 h; 7–7.9 h (reference))
  ○ (<5 h; >7 h; 5–7 h (reference))
- Total sleep duration (<6 h; >8 h; 6–8 h (reference))
- Total sleep duration (⩽5 h; 5.1–6.9 h; ⩾8 h; 7–7.9 h (reference))
- Daytime napping (regular versus irregular/no napping (reference))
- Regular difficulties falling asleep (yes/no)
- Regular difficulties maintaining sleep (yes/no)
- Regular early morning arousal (yes/no)
- Any regular sleep disturbance (yes/no)

In further linear regression analyses with weight change between T0 and T1 as the outcome, the exposure variable (T0) was generated by splitting each category of sleep duration into two (with or without report of any regular sleep disturbance) using normal sleep duration (6–8 h or 7–<8 h, respectively) without regular sleep disturbances as the category of reference.

To estimate weight change (and change of waist circumference, respectively) between T1 and T2 from patterns of sleep characteristics at T0 and at T1, we used a dataset with complete data on sleep duration at T0 and T1, covariates at T1, and weight at T0, T1 and T2 leaving a total of 2837 participants. In linear regression analyses, weight change (and change of waist circumference, respectively) between T1 and T2 was estimated from patterns of sleep characteristics (sleep characteristic present at T0 and T1, only at T1, only at T0, neither at T0 nor at T1 (reference)).

We used the software DAGitty to identify a minimally sufficient adjustment set.^21^ The minimal adjustment set included age (continuous), sex, weight at baseline, alcohol intake (g day^−1^), smoking (current, former, never), accordance with dietary guidelines (low, medium, high), physical activity (T0: metabolic equivalents per week; T1: amount of sport assessed as ⩾2 h, 1–2 h, 0–<1 h), school education (low/medium/high), marital stage (married and living together (yes/no)), subjective health status (‘very good/good' versus ‘satisfactory/poor/very poor') and stress (continuous). The minimal adjustment set additionally included sleep duration for siesta as the exposure of interest, and regular sleep disturbances for sleep duration as the exposure of interest. Coffee consumption, depression and hypertension were included in the directed acyclic graph but were not selected for the minimal adjustment set. In the linear regression analyses, adjustment for potential confounders was performed for age, sex and weight at T0 (and weight change between T0 and T1, respectively) (model 1), and for the minimally sufficient adjustment set (model 2).

We conducted several sensitivity analyses. First, analyses were confined to persons who did not report chronic illness (that is, cancer, stroke, cardiovascular disease). The rationale for this analysis was twofold: there is an indication that associations between sleep characteristics and metabolic disorders may be different in persons with and without chronic disease like cancer, stroke and cardiovascular diseases; and, moreover, confounding may be more of a problem in chronic ill persons. Second, sex-stratified analyses were done for some selected sleep variables. Third, some selected analyses were confined to subjects with BMI at baseline <30 kg m^−^^2^. Fourth, we repeated analyses of associations between sleep characteristics at T0 and weight change between T0 and T1 for two strata: the first stratum included subjects working at least 15 h week^−1^ at baseline without experience of night shifts, the second stratum included subjects working less than 15 h week^−1^. Fifth, we stratified some analyses on sleep characteristics at baseline and weight change between T0 and T1 by weight change (⩾/<5%).

All statistical analyses were performed using SAS version 9.4 (SAS Institute Inc., Cary, NC, USA). We are calculating and reporting confidence intervals to assess the precision of our estimates because our goal is estimation and not significance testing.^22, 23^ We wish to avoid publication bias by preferential reporting of significant results. Instead, we judge the value of our estimates by their precision and validity.

## Results

Overall, 457 (12.2%) participants reported <6 h nocturnal sleep, 244 (6.5%) participants reported more than 8 h nocturnal sleep and 80% subjects fell in the range of 6–8 h of night sleep (Table 1). In each category of nocturnal sleep duration, about one in six subjects took a regular daytime nap. Regular difficulties falling asleep and regular early morning arousal were common in short sleepers, but rare in persons with normal and long duration of night sleep. The precise distribution of nocturnal sleep duration is shown in Supplementary Table 1.

**Figure 1:**
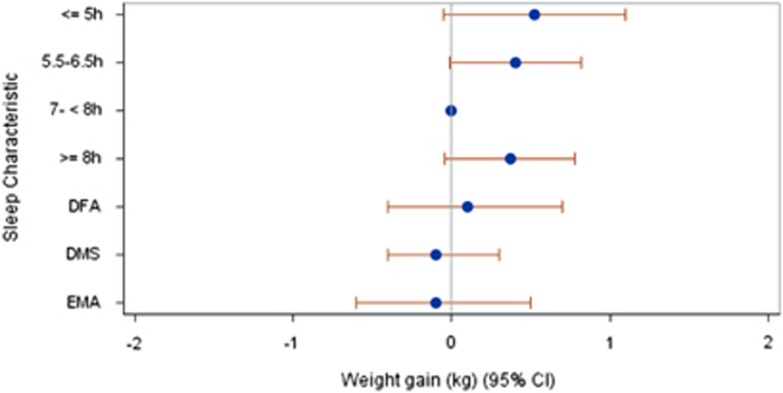
Nocturnal sleep duration (reference: 7–<8 h) and regular sleep disorders, respectively, at baseline and weight gain between baseline and first follow-up visit. Results from fully adjusted linear regression models: the Heinz Nixdorf Recall study (*N*=3751). DFA, regular difficulties falling asleep; DMS, regular difficulties maintaining sleep; EMA, regular early morning arousal.

Short or long nocturnal sleep at baseline (T0) was associated with less than one kilogram weight gain during the first period of follow-up (T0 to T1) regardless of varying definitions of short (<6, <5, ⩽5 h) and long (>7, >8, ⩾8 h) sleep (Figure 1; Table 2). Moreover, daytime napping and regular sleep disturbances at T0 were associated with weight gain close to zero between T0 and T1.

Short sleepers with any regular sleep disturbances at T0 (and long sleepers with any regular sleep disturbances at T0, respectively) experienced weight gain<1 kg during T0 and T1 compared with persons with normal sleep duration (6–8, or 7–<8 h) without regular sleep disturbances (Table 3).

Selecting subjects who did not report regular sleep disturbances at T0 and at T1 as the reference category, subjects reporting regular sleep disturbances at T0 and T1 gained<0.5 kg weight during the second follow-up (T1 to T2) (Table 4). Likewise, daytime nappers at T0 and T1 gained 0.1 kg more weight between T1 and T2 than subjects who did not take regular daytime naps at T0 and T1. In addition, short (<6 h) and long (>8 h) nocturnal sleep at T0 and T1, respectively, were associated with weight gain <0.5 kg between T1 and T2 compared with the 6–8 h nocturnal sleep at T0 and T1 as the reference. Analogously, all analyses on the associations between sleep characteristics at T0 and T1 and changes of waist circumference between T1 and T2 showed an increase in waist circumference which was lower than 1 cm (Table 5).

Virtually the same results were observed when analyses were confined to subjects who did not report any chronic diseases (Supplementary Table 2). Moreover, only small sex differences (⩽0.6 kg) in the association between sleep characteristics and weight gain were seen. In subjects with BMI at baseline <30 kg m^−2^, weight gain was not stronger than in the whole population (Supplementary Table 3). When analyses of associations between sleep characteristics at T0 and weight change between T0 and T1 were stratified by occupational status, only little weight gain was observed in the two strata (cf. Supplementary Table 4). In further stratified analyses, associations between sleep characteristics at baseline and weight change between T0 and T1 were small for subjects with weight change ⩾5% and subjects with weight change <5% (cf. Supplementary Table 5).

## Discussion

In the present study on older adults, we did not find that self-reported sleep duration and sleep disturbances are associated with clinically relevant weight change or change of waist circumference over a 5-year follow-up. In particular, subjects who reported short (or long) sleep or regular sleep disturbances at two points in time did not gain more weight than subjects consistently reporting normal sleep characteristics. Moreover, we found no evidence that subjects characterized by a combination of short (or long) sleep duration and regular sleep disturbances, gained more weight than persons with normal sleep duration who reported no regular sleep disturbances. Furthermore, regular daytime nappers were not at an increased risk of weight gain.

### Methodical considerations

In most studies published so far, sleep characteristics were assessed only at one point in time. However, sleep characteristics may change over time. For example, in the Spanish Pizarra Study, nocturnal sleep duration was measured at baseline, at 6- and at 11-year follow-up, and correlations between these measurements were rather poor.^24^ Therefore, it is worthwhile assessing sleep characteristics at two study points and to assess whether subjects with short (or long) sleep duration (or sleep disturbances) at both study points are at a particular risk of weight gain. In the Pizarra Study, subjects with short sleep duration at two or three study points had a higher incidence of obesity. However, in these analyses, the 11-year time period used to measure sleep duration completely overlapped with the follow-up for assessment of incident obesity thereby ignoring the potential latency of the effect of sleep characteristics on weight.^24^ In a Swedish 10-year follow-up study, younger women with unchanged short sleep duration measured at baseline and at follow-up were at a much higher risk of obesity than women with short sleep duration only at one study point.^25^ But in this study, too, the time period for measurement of the exposure and the time of follow-up were identical. On the contrary, in our study, sleep duration was assessed at baseline and at the first follow-up visit, and, thus, clearly preceded the follow-up time for observation of weight change, which covered the time period from first to second follow-up visit.

In previous studies, various sleep durations were selected as the reference category, and definitions of short and long duration of nocturnal sleep varied widely.^8^ In our study, we selected various sleep durations as a reference category or as short or long sleep duration, but this had virtually no influence on the null effects in our analyses.

Some authors have questioned the validity of self-reported sleep characteristics.^11, 12, 26^ Comparisons of self-reported and objective measures of sleep duration have revealed strong discrepancies.^27, 28^ In the cross-sectional Rotterdam Study of elderly subjects, a U-shaped association with BMI and obesity was found for sleep duration measured by actigraphy, but not for self-reported sleep duration.^26^ As an explanation, the authors of this study assume that sleep disturbances occur more frequently in the elderly who are, however, often unaware of these disturbances, and, thus, give less valid self-reports of their sleep duration. Yet, in two longitudinal studies objective sleep duration was not associated with an increased risk of obesity.^6, 29^ Vgontzas *et al.*^6^ reported that associations between self-reported short sleep duration and incident obesity were strongly attenuated after adjustment for subjective sleep disturbances and emotional stress. This suggests that self-reported sleep duration is an indicator not only of objective sleep duration, but also of other psychological and health related factors. Thus, objective and self-reported sleep duration may at least partly measure different phenomena.

### Daytime napping and weight gain

To our knowledge, the association between daytime napping and weight gain has only been investigated once so far.^7^ In the Spanish SUN Mediterranean Cohort, persons with a 30 min daily siesta had a reduced odds of incident obesity (OR=0.67, 95% CI: 0.46–0.96 compared with non-nappers). For longer siestas, this odds reduction was smaller (OR=0.92, 95% CI: 0.72–1.18 for 31–59 min of daily siesta; OR=0.78, 95% CI: 0.51–1.19 for ⩾60 min of daily siesta). In our own study, regular daytime napping was not associated with weight gain, and results for total sleep duration including nocturnal and daytime sleep were virtually the same as results for nocturnal sleep alone. Results from a country with a long cultural habit of siesta may not be fully comparable with results from Germany. Anyhow, the Spanish and our own study give evidence that daytime napping is not a risk factor for weight gain.

### The role of age in associations between sleep duration and weight gain

Our result that sleep duration is not associated with weight gain in older adults is in line with several other studies. In cross-sectional analyses, Hasler *et al.*^9^ found strong associations between short sleep duration and obesity only at ages 27, 29 and 34, but not at age 40. Some longitudinal studies also indicated that sleep duration is not associated with weight change in older adults. In a Swedish study, associations between habitual short (or long) sleep duration and obesity were found in younger (<40 years), but not in older women (⩾40 years).^25^ In the Penn State Cohort, self-reported short sleep was associated with incident obesity in young and middle-aged adults, but not in adults older than 40 years.^6^ Ford *et al.*^30^ speculate that potential confounders like comorbidities and medication use may play a minor role in the young. Magee *et al.*^1^ suggest that the impact of short sleep on weight gain diminishes after transition to short sleep which might imply that the impact of sleep duration on body weight alters in the course of life. Nevertheless, no convincing explanation for the lack of an association in older subjects has been given so far, and in her comprehensive review, Knutson simply states that these age differences require further examination.^31^

### Public health relevance of the results

In a public health perspective, it can be concluded from our study that sleep is not a risk factor of weight gain in older adults. Even for other studies reporting associations between short (or long) sleep duration and weight gain mainly in younger adults, it should be stressed that reported changes of weight or BMI were often clinically negligible, in particular when related to the often many years of follow-up. To give some examples, the 1-year change in BMI was 0.11 kg m^−^^2^ (95% CI: 0.05–0.18) for 5-h sleep duration in younger Japanese workers;^32^ weight change was 0.64 kg (95% CI: 0.17–1.12) over an average of 7.5 years in older US men;^5^ in the Nurses Health Study, women sleeping at most 5 h per night gained additional 1.14 kg over 16 years.^33^ This leads us to agree with Horne who stated that the weak association between short sleep duration and weight gain does not justify intervention directed at a prolongation of sleep, and who pointed out that exercising takes much less time to lose weight than prolonging night sleep from 5 to 7 h.^34^

### Strengths and limitations

Our study has several strengths. It is a large prospective, population-based cohort study. Sleep quality and quantity were assessed comprehensively, including duration and frequency of daytime napping. We had three study points which enabled us to assess associations between time patterns of sleep characteristics and subsequent weight gain. Moreover, we did several sensitivity analyses which all were in line with our main results of no weight gain in older subjects with extreme sleep duration or sleep disorders. In particular, results were virtually the same for those with and without strong weight gain. Thus, limited overall weight gain does not explain that sleep characteristics are barely associated with weight gain in our study.

There are some limitations to consider. First, sleep characteristics have not been measured objectively by actigraphy, polygraphy, or polysomnography. Second, in the cross-sectional Study of Osteoporotic Fractures, variability in nocturnal sleep duration and daytime napping was shown to be associated with obesity independent of mean sleep duration.^35^ In our study, we were not able to investigate the impact of variability of sleep characteristics on weight change. Third, we did not distinguish between work days and non-work days in our questions on sleep duration and napping. However, our main result of no weight gain in people with long/short sleep duration or with regular sleep disturbances was also found in subjects who were not employed and for whom the distinction between work days and weekend should be of little relevance. Fourth, in analyses on associations between sleep characteristics (measured at T0 and T1) and subsequent weight change (between T1 and T2), one covariate (stress) was measured at T0, but not at T1. However, in our study, associations were virtually null in the age–sex adjusted models, and further adjustment had almost no effect on effect estimates.

## Conclusion

Our study provides no evidence that self-reported sleep duration and sleep disturbances are associated with clinically relevant weight gain in the older population. Contrary to previous studies, we addressed some specific methodical issues (measurement of sleep characteristics at two points in time; consideration of daytime napping; varying definitions of short and long sleep duration). From our and other studies in older people, we conclude that interventions to improve night sleep are not an adequate mean to avoid weight increase in older people.

## Figures and Tables

**Table 1:** Baseline characteristics stratified by duration of nocturnal sleep: the Heinz Nixdorf Recall study (*N*=3751)

	*Duration of nocturnal sleep*		
	*<6 h*	*6–8 h*	*>8 h*
*N*	457	3050	244
Age (years)	58.6±7.3	58.7±7.6	62.2±6.7
Men (%)	41.4	52.0	50.0
Weight (kg)	79.3±15.5	79.2±14.7	80.0±16.2
Alcohol consumption (g day^−1^)	7.6±14.6	8.5±14.8	9.7±17.7
Smoking (current/former/never) (%)	23.2/35.0/41.8	21.3/36.7/42.0	26.2/32.4/41.4
Accordance with dietary guidelines (low/medium/high) (%)	38.1/31.7/30.2	37.5/35.3/27.2	38.9/32.0/29.1
Metabolic equivalents/week	39.4±42.9	45.2±46.2	51.2±52.0
School education (low/medium/high) (%)	63.0/18.8/18.2	56.3/19.1/24.6	72.5/13.1/14.3
Marital stage (married) (%)	70.9	77.3	81.2
Subjective health (very good/good) (%)	33.7	52.9	48.8
Stress^a^	1.5±0.6	1.4±0.4	1.3±0.4
Regular daytime napping (%)	17.1	15.1	16.4
Regular difficulties falling asleep (%)	30.3	5.9	2.9
Regular difficulties maintaining sleep (%)	56.0	32.7	32.0
Regular early morning arousal (%)	32.4	7.1	2.5
Any regular sleep disturbance (%)^b^	64.8	36.7	33.6
Occupational status (employed) (%)^c^	42.9	44.0	17.2
Weight change between T0 and T1 (kg)	1.0±5.6	0.6±5.0	1.0±5.3
Weight change between T0 and T2 (kg)	1.7±6.6	1.0±6.5	0.8±6.5
Change of waist circumference between T0 and T1 (cm)	2.4±6.9	2.3±6.5	2.0±6.2
Change of waist circumference between T0 and T2 (cm)	3.9±8.1	3.6±7.5	2.8±7.3

Abbreviations: T0, time of baseline visit; T1, time of second visit; T2, time of third visit.

a Higher values mean higher levels of stress.

b Regular difficulties falling asleep, regular difficulties maintaining sleep or regular early morning arousal.

c Subjects are considered as occupied if they work at least 15 h week^−1^.

Values are expressed as mean±s.d., or proportion (%).

**Table 2:** Linear regression models analyzing associations between sleep characteristics at T0 and weight change between T0 and T1 (regression coefficients β with 95% confidence intervals (CI)): the Heinz Nixdorf Recall study (*N*=3751)

	N	*Mean weight change (kg)*	*Model 1 β (95% CI) (kg)*	*Model 2 β (95% CI) (kg)*
*Duration of night sleep*				
<6 h	457	0.99	0.4 (−0.1; 0.9)	0.3 (−0.2; 0.8)
>8 h	244	0.95	0.7 (0.0; 1.4)	0.7 (0.0; 1.3)
6–8 h (ref)	3050	0.61	0	0
				
*Duration of night sleep*				
<5 h	148	1.16	0.6 (−0.2; 1.5)	0.6 (−0.2; 1.4)
5–5.9 h	309	0.91	0.3 (−0.3; 0.8)	0.2 (−0.4; 0.8)
>8 h	244	0.95	0.7 (0.0; 1.4)	0.7 (0.0; 1.3)
6–8 h (ref)	3050	0.61	0	0
				
*Duration of night sleep*				
⩽5 h	402	1.02	0.6 (0.0; 1.2)	0.5 (−0.1; 1.1)
5.1–6.9 h	972	0.88	0.5 (0.0; 0.9)	0.4 (0.0; 0.8)
⩾8 h	1098	0.60	0.4 (0.0; 0.8)	0.4 (0.0; 0.8)
7–7.9 h (ref)	1279	0.48	0	0
				
*Duration of night sleep*				
<5 h	148	1.16	0.7 (−0.2; 1.5)	0.6 (−0.3; 1.5)
>7 h	1260	0.64	0.2 (−0.1; 0.6)	0.2 (−0.1; 0.6)
5–7 h (ref)	2343	0.67	0	0
				
*Total sleep*				
<6 h	424	1.05	0.4 (−0.1; 0.9)	0.4 (−0.1; 0.9)
>8 h	718	0.46	0.2 (−0.2; 0.6)	0.2 (−0.2; 0.6)
6–8 h (ref)	2605	0.68	0	0
				
*Total sleep*				
⩽5 h	259	1.27	0.8 (0.1; 1.5)	0.7 (0.1; 1.4)
5.1–6.9 h	1020	0.86	0.4 (−0.1; 0.8)	0.3 (−0.1; 0.7)
⩾8 h	1183	0.55	0.3 (−0.1; 0.7)	0.3 (−0.1; 0.7)
7–7.9 h (ref)	1285	0.54	0	0
				
*Daytime napping*				
Regular	579	0.06	−0.3 (−0.7; 0.2)	−0.3 (−0.7; 0.2)
No/irregular (ref)	3171	0.79	0	0
				
*Regular difficulties falling asleep*				
Yes	324	0.86	0.3 (−0.3; 0.9)	0.1 (−0.4; 0.7)
No (ref)	3425	0.66	0	0
				
*Regular difficulties maintaining sleep*				
Yes	1332	0.44	0.0 (−0.4; 0.3)	−0.1 (−0.4; 0.3)
No (ref)	2419	0.81	0	0
				
*Regular early morning arousal*				
Yes	367	0.65	0.0 (−0.5; 0.5)	−0.1 (−0.6; 0.5)
No (ref)	3370	0.69	0	0
				
*Any regular sleep disturbance*^a^				
Yes	1497	0.45	0.0 (−0.4; 0.3)	−0.1 (−0.4; 0.3)
No (ref)	2254	0.83	0	0

Abbreviations: T0, time of baseline study; T1, time of second visit.

a Regular difficulties falling asleep or regular difficulties maintaining sleep or regular early morning arousal.

Model 1: adjusted for age, sex and weight at baseline.

Model 2: adjusted for age, sex, weight at baseline, alcohol intake, smoking, accordance with dietary guidelines, metabolic equivalents/week, education, marital stage, subjective health, stress. For daytime napping as the exposure variable, additional adjustment for sleep duration; for sleep duration as the exposure variable, additional adjustment for any regular sleep disturbances.

**Table 3:** Linear regression models analyzing associations between patterns of sleep duration and prevalence of regular sleep disturbances at T0 and weight change between T0 and T1 (regression coefficients β with 95% CIs): the Heinz Nixdorf Recall study

	*Any regular sleep disturbance*^a^	N	*Mean weight change (kg)*	*Model 1 β (95% CI) (kg)*	*Model 2 β (95% CI) (kg)*
*Nocturnal sleep duration*					
<6 h	Yes	296	0.79	0.3 (−0.4; 0.9)	0.1 (−0.5; 0.8)
<6 h	No	161	1.36	0.6 (−0.2; 1.4)	0.5 (−0.3; 1.3)
>8 h	Yes	82	0.65	0.5 (−0.6; 1.6)	0.5 (−0.6; 1.6)
>8 h	No	162	1.11	0.8 (0.0; 1.6)	0.7 (−0.1; 1.5)
6–8 h	Yes	1119	0.35	0.0 (−0.4; 0.4)	0.0 (−0.4; 0.4)
6–8 h	No	1931	0.76	0	0
					
*Total sleep duration*					
<6 h	Yes	279	0.85	0.2 (−0.4; 0.9)	0.1 (−0.5; 0.8)
<6 h	No	145	1.43	0.6 (−0.2; 1.5)	0.6 (−0.3; 1.4)
>8 h	Yes	286	0.42	0.3 (−0.4; 0.9)	0.3 (−0.4; 0.9)
>8 h	No	432	0.49	0.0 (−0.5; 0.6)	0.0 (−0.5; 0.6)
6–8 h	Yes	929	0.34	−0.1 (−0.6; 0.3)	−0.2 (−0.6; 0.2)
6–8 h	No	1676	0.86	0	0
					
*Nocturnal sleep duration*					
⩽5 h	Yes	267	0.76	0.3 (−0.3; 1.0)	0.2 (−0.5; 0.9)
⩽5 h	No	135	1.53	0.9 (0.0; 1.8)	0.8 (−0.1; 1.7)
5.1–6.9 h	Yes	376	0.74	0.4 (−0.2; 1.1)	0.4 (−0.2; 1.0)
5.1–6.9 h	No	596	0.97	0.4 (−0.2; 0.9)	0.3 (−0.2; 0.8)
⩾8 h	Yes	407	0.38	0.4 (−0.3; 1.0)	0.3 (−0.3; 0.9)
⩾8 h	No	691	0.73	0.3 (−0.2; 0.8)	0.3 (−0.2; 0.8)
7–7.9 h	Yes	447	0.09	−0.2 (−0.7; 0.4)	−0.2 (−0.8; 0.4)
7–7.9 h	No	832	0.69	0	0

Abbreviations: T0, time of baseline study; T1, time of second visit.

a Regular difficulties falling asleep or regular difficulties maintaining sleep or regular early morning arousal.

Model 1: adjusted for age, sex and weight at baseline.

Model 2: adjusted for age, sex, weight at baseline, alcohol intake, smoking, accordance with dietary guidelines, metabolic equivalents/week, education, marital stage, subjective health, stress.

**Table 4:** Linear regression models analyzing associations between patterns of sleep characteristics at T0 and T1 and weight change between T1 and T2 (regression coefficients β with 95% CIs): the Heinz Nixdorf Recall study

	N	*Mean weight change (kg)*	*Model 1 β (95% CI) (kg)*	*Model 2 β (95% CI) (kg)*
*Regular difficulties falling asleep*				
At T0 and at T1	96	0.13	0.1 (−0.9, 1.1)	0.1 (−0.9, 1.1)
Only at T1	141	−0.22	−0.5 (−1.4, 0.3)	−0.5 (−1.4, 0.3)
Only at T0	134	−0.05	−0.3 (−1.1, 0.6)	−0.3 (−1.1, 0.6)
Neither at T0 nor at T1 (ref)	2463	0.27	0	0
				
*Regular difficulties maintaining sleep*				
At T0 and at T1	621	0.10	0.1 (−0.3, 0.6)	0.1 (−0.4, 0.6)
Only at T1	483	0.13	0.0 (−0.5, 0.5)	0.0 (−0.5, 0.5)
Only at T0	345	0.28	0.3 (−0.2, 0.9)	0.3 (−0.3, 0.9)
Neither at T0 nor at T1 (ref)	1387	0.32	0	0
				
*Regular early morning arousal*				
At T0 and at T1	72	0.47	0.2 (−1.0, 1.3)	0.2 (−1.0, 1.3)
Only at T1	222	0.84	0.7 (0.0, 1.3)	0.7 (0.0, 1.3)
Only at T0	186	−0.34	−0.5 (−1.2, 0.3)	−0.5 (−1.2, 0.3)
Neither at T0 nor at T1 (ref)	2334	0.23	0	0
				
*Any regular sleep disturbance*^a^				
At T0 and at T1	725	0.15	0.1 (−0.4, 0.5)	0.1 (−0.4, 0.5)
Only at T1	484	0.10	−0.1 (−0.7, 0.4)	−0.2 (−0.7, 0.4)
Only at T0	361	−0.01	−0.1 (−0.7, 0.5)	−0.1 (−0.7, 0.5)
Neither at T0 nor at T1 (ref)	1267	0.40	0	0
				
*Regular napping*				
At T0 and at T1	237	−0.13	0.1 (−0.5, 0.8)	0.1 (−0.6, 0.8)
Only at T1	182	−0.50	−0.3 (−1.1, 0.4)	−0.3 (−1.1, 0.4)
Only at T0	149	−0.09	−0.2 (−1.0, 0.6)	−0.2 (−1.0, 0.6)
Neither at T0 nor at T1 (ref)	2268	0.35	0	0
				
*Sleep duration <6* h				
At T0 and at T1	178	0.72	0.4 (−0.3, 1.2)	0.4 (−0.3, 1.2)
Only at T1	181	0.02	−0.4 (−1.1, 0.4)	−0.4 (−1.1, 0.4)
Only at T0	150	−0.28	−0.4 (−1.2, 0.4)	−0.4 (−1.3, 0.4)
Neither at T0 nor at T1 (ref)	2053	0.32	0	0
				
*Sleep duration >8 h*				
At T0 and at T1	67	−1.08	−0.8 (−2.0, 0.4)	−0.8 (−2.0. 0.4)
Only at T1	105	−0.12	−0.1 (−1.1. 0.9)	−0.1 (−1.1, 0.8)
Only at T0	87	0.2	0.1 (−1.0, −1.1)	0.1 (−1.0. 1.1)
Neither at T0 nor at T1 (ref)	2053	0.32	0	0

Abbreviations: T0, time of baseline study; T1, time of second visit; T2, time of third visit.

a Regular difficulties falling asleep or regular difficulties maintaining sleep or regular early morning arousal.

Model 1: adjusted for age, sex and weight change between T0 and T1.

Model 2: adjusted for age, sex, weight change between T0 and T1, alcohol intake, smoking, accordance with dietary guidelines, physical activity, education, marital stage, subjective health, stress.

**Table 5:** Linear regression models analyzing associations between patterns of sleep characteristics at T0 and T1 and change of waist circumference between T1 and T2 (regression coefficients β with 95% Cls): the Heinz Nixdorf Recall study

	N	*Mean change of waist circ. (cm)*	*Model 1 β (95% CI) (cm)*	*Model 2 β (95% CI) (cm)*
*Regular difficulties falling asleep*				
At T0 and at T1	96	1.31	0.2 (−1.1, 1.6)	0.1 (−1.3, 1.5)
Only at T1	141	1.06	−0.2 (−1.4, 0.9)	−0.3 (−1.4, 0.9)
Only at T0	134	0.77	−0.5 (−1.6, 0.7)	−0.6 (−1.8, 0.6)
Neither at T0 nor at T1 (ref)	2461	1.34	0	0
				
*Regular difficulties maintaining sleep*				
At T0 and at T1	620	1.31	0.2 (−0.5, 0.8)	0.1 (−0.5, 0.8)
Only at T1	483	0.86	−0.4 (−1.1, 0.3)	−0.4 (−1.1, 0.3)
Only at T0	345	1.51	0.4 (−0.4, 1.2)	0.3 (−0.5, 1.1)
Neither at T0 nor at T1 (ref)	1386	1.40	0	0
				
*Regular early morning arousal*				
At T0 and at T1	72	1.29	0.0 (−1.6, 1.6)	0.0 (−1.6, 1.6)
Only at T1	222	1.59	0.4 (−0.6, 1.3)	0.3 (−0.6, 1.3)
Only at T0	186	1.12	−0.1 (−1.1, 0.9)	−0.1 (−1.1, 0.9)
Neither at T0 nor at T1 (ref)	2332	1.29	0	0
				
*Any regular sleep disturbance*^a^				
At T0 and at T1	724	1.36	0.1 (−0.5, 0.7)	0.0 (−0.6, 0.7)
Only at T1	484	0.78	−0.6 (−1.3, 0.1)	−0.6 (−1.3, 0.1)
Only at T0	361	1.15	−0.2 (−0.9, 0.6)	−0.2 (−1.0, 0.6)
Neither at T0 nor at T1 (ref)	1266	1.51	0	0
				
*Regular napping*				
At T0 and at T1	237	0.80	−0.2 (−1.1, 0.7)	−0.2 (−1.2, 0.7)
Only at T1	182	1.18	0.1 (−0.9, 1.2)	0.1 (−0.9, 1.1)
Only at T0	149	1.01	−0.2 (−1.3, 0.9)	−0.3 (−1.4, 0.8)
Neither at T0 nor at T1 (ref)	2266	1.38	0	0
				
*Sleep duration <6* h				
At T0 and at T1	178	1.58	0.4 (−0.7, 1.4)	0.3 (−0.7, 1.3)
Only at T1	181	1.70	0.3 (−0.7; 1.4)	0.4 (−0.7; 1.4)
Only at T0	150	0.89	−0.2 (−1.3, 0.9)	−0.3 (−1.4, 0.9)
Neither at T0 nor at T1 (ref)	2052	1.34	0	0
				
*Sleep duration >8* h				
At T0 and at T1	67	1.44	0.6 (−1.1, 2.2)	0.6 (−1.1, 2.2)
Only at T1	105	0.43	−0.7 (−2.0, 0.7)	−0.7 (−2.0, 0.7)
Only at T0	86	0.92	−0.3 (−1.7, 1.2)	−0.3 (−1.8, 1.1)
Neither at T0 nor at T1 (ref)	2052	1.34	0	0

Abbreviations: T0, time of baseline study; T1, time of second visit; T2, time of third visit.

a Regular difficulties falling asleep, regular difficulties maintaining sleep or regular early morning arousal.

Model 1: adjusted for age, sex and weight change between T0 and T1.

Model 2: adjusted for age, sex, weight change between T0 and T1, alcohol intake, smoking, accordance with dietary guidelines, physical activity, education, marital stage, subjective health, stress.
