# Supplementary material for: Associations between sleep characteristics and weight gain in an older population: results of the Heinz Nixdorf Recall Study
Source: Nutr Diabetes. 2016 Aug 15;6(8):e225–. doi: 10.1038/nutd.2016.32 (PMC5022146; doi:10.1038/nutd.2016.32)
Supplement: Supplementary Table 1 [file nutd201632x1.docx]

**Supplementary table 1**

Distribution of reported duration of nocturnal sleep

(baseline assessment (2000 – 2003)): The Heinz Nixdorf Recall study

| Sleep duration (hours) | N | per cent |
| --- | --- | --- |
| ≤ 3 | 33 | 0.9 |
| 3.5 | 9 | 0.2 |
| 4 | 80 | 2.1 |
| 4.5 | 26 | 0.7 |
| 5 | 254 | 6.8 |
| 5.5 | 55 | 1.5 |
| 6 | 721 | 19.2 |
| 6.083 | 3 | 0.1 |
| 6.5 | 193 | 5.2 |
| 7 | 1117 | 29.8 |
| 7.083 | 1 | 0.03 |
| 7.5 | 160 | 4.2 |
| 8 | 854 | 22.8 |
| 8.03 | 1 | 0.03 |
| 8.25 | 1 | 0.03 |
| 8.5 | 28 | 0.8 |
| 9 | 142 | 3.8 |
| 9.5 | 7 | 0.2 |
| 10 | 61 | 1.6 |
| 11 | 2 | 0.05 |
| 12 | 2 | 0.05 |
