# Supplementary material for: Associations between sleep characteristics and weight gain in an older population: results of the Heinz Nixdorf Recall Study
Source: Nutr Diabetes. 2016 Aug 15;6(8):e225–. doi: 10.1038/nutd.2016.32 (PMC5022146; doi:10.1038/nutd.2016.32)
Supplement: Supplementary Table 2 [file nutd201632x2.docx]

**Supplementary table 2.**

Linear regression models analyzing associations between sleep characteristics at T0 and weight change between T0 and T1 (regression coefficients with 95% confidence intervals): stratified by sex; only for subjects without chronic illness ^a^

|  | **Sex** | | **No chronic illness** |
| --- | --- | --- | --- |
|  | Men | Women |  |
|  | 1,897 | 1,854 | 3,261 |
|  | ß (95% CI) (kg) | ß (95% CI) (kg) | ß (95% CI) (kg) |
| **Nocturnal sleep duration** |  |  |  |
| < 6 h | 0.4 (-0.3, 1.2) | 0.1 (-0.5, 0.8) | 0.5 (-0.1, 1.0) |
| > 8 h | 0.9 (0.0, 1.8) | 0.4 (-0.5, 1.4) | 0.8 (0.1, 1.5) |
| 6 – 8 h (ref) | 0 | 0 | 0 |
| **Nocturnal sleep duration** |  |  |  |
| < 5 h | 0.9 (-0.4, 2.2) | 0.3 (-0.7, 1.4) | 0.8 (-0.1, 1.7) |
| 5 - < 6 h | 0.3 (-0.6, 1.1) | 0.0 (-0.7, 0.8) | 0.4 (-0.2, 1.0) |
| > 8 h | 0.9 (0.0, 1.8) | 0.4 (-0.5, 1.4) | 0.8 (0.1, 1.5) |
| 6 – 8 h (ref) | 0 | 0 | 0 |
| **Any regular sleep disturbance ^b^** |  |  |  |
| yes | -0.1 (-0.6, 0.3) | 0.0 (-0.5, 0.5) | 0.0 (-0.4, 0.3) |
| no | 0 | 0 | 0 |

T0: time of baseline visit; T1: time of second visit

^a^ Adjusted for age, sex, weight change between T0 and T1, alcohol intake, smoking, accordance with dietary guidelines, physical activity, education, marital stage, subjective health, stress

^b^ Regular difficulties falling asleep, regular difficulties maintaining sleep or regular early morning arousal
