# Supplementary material for: Associations between sleep characteristics and weight gain in an older population: results of the Heinz Nixdorf Recall Study
Source: Nutr Diabetes. 2016 Aug 15;6(8):e225–. doi: 10.1038/nutd.2016.32 (PMC5022146; doi:10.1038/nutd.2016.32)
Supplement: Supplementary Table 3 [file nutd201632x3.docx]

**Supplementary table 3.**

Linear regression models analyzing associations between sleep characteristics at T0 and weight change between T0 and T1 for subjects with BMI at baseline < 30 kg/m^2^ (regression coefficients with 95% confidence intervals)

|  | **N** | **Mean weight change (kg)** | **Model 1**  **ß (95% CI) (kg)** | **Model 2**  **ß (95% CI) (kg)** |
| --- | --- | --- | --- | --- |
| **Duration of night sleep** |  |  |  |  |
| < 6 h | 320 | 0.94 | 0.1 (-0.4; 0.6) | 0.0 (-0.5; 0.5) |
| > 8 h | 178 | 1.13 | 0.6 (-0.1; 1.2) | 0.5 (-0.1; 1.2) |
| 6 – 8 h (ref) | 2,293 | 0.83 | 0 | 0 |
| **Duration of night sleep** |  |  |  |  |
| ≤ 5 h | 284 | 1.02 | 0.4 (-0.2; 0.9) | 0.3 (-0.3; 0.9) |
| 5.1 – 6.9 h | 707 | 1.08 | 0.4 (0.0; 0.8) | 0.3 (-0.1; 0.8) |
| ≥ 8 h | 816 | 0.82 | 0.4 (0.0; 0.8) | 0.4 (0.0; 0.8) |
| 7 – 7.9 h (ref) | 984 | 0.70 | 0 | 0 |
| **Total sleep** |  |  |  |  |
| < 6 h | 300 | 1.01 | 0.2 (-0.3; 0.7) | 0.1 (-0.4; 0.7) |
| > 8 h | 514 | 0.81 | 0.3 (-0.1; 0.8) | 0.3 (-0.1; 0.8) |
| 6 – 8 h (ref) | 1,973 | 0.85 | 0 | 0 |
| **Daytime napping** |  |  |  |  |
| Regular | 425 | 0.44 | -0.1 (-0.5; 0.4) | -0.1 (-0.5; 0.4) |
| No / irregular (ref) | 2,365 | 0.94 | 0 | 0 |
| **Any regular sleep disturbance ^a^** |  |  |  |  |
| yes | 1,059 | 0.61 | -0.1 (-0.4; 0.2) | -0.1 (-0.5; 0.2) |
| no (ref) | 1,732 | 1.02 | 0 | 0 |

T0: time of baseline study; T1: time of second visit

Model 1: adjusted for age, sex and weight at baseline

Model 2: adjusted for age, sex, weight at baseline, alcohol intake, smoking, accordance with dietary guidelines, metabolic equivalents / week, education, marital stage, subjective health, stress. For daytime napping as the exposure variable, additional adjustment for sleep duration; for sleep duration as the exposure variable, additional adjustment for any regular sleep disturbances.
