# Supplementary material for: Associations between sleep characteristics and weight gain in an older population: results of the Heinz Nixdorf Recall Study
Source: Nutr Diabetes. 2016 Aug 15;6(8):e225–. doi: 10.1038/nutd.2016.32 (PMC5022146; doi:10.1038/nutd.2016.32)
Supplement: Supplementary Table 4 [file nutd201632x4.docx]

**Supplementary table 4a.**

Linear regression models analyzing associations between sleep characteristics at T0 and weight change between T0 and T1 (regression coefficients with 95% confidence intervals): The Heinz Nixdorf Recall study.

Only subjects working at least 15 hours / week at baseline without night shifts in their former professional life (N=1,245).

|  | **N** | **Mean weight change (kg)** | **Model 1**  **ß (95% CI) (kg)** | **Model 2**  **ß (95% CI) (kg)** |
| --- | --- | --- | --- | --- |
| **Duration of night sleep** |  |  |  |  |
| < 6 h | 135 | 1.73 | 0.7 (-0.2; 1.6) | 0.5 (-0.5; 1.4) |
| > 8 h | 32 | 0.99 | -0.2 (-2.0; 1.6) | -0.3 (-2.1; 1.5) |
| 6 – 8 h (ref) | 1,078 | 1.15 | 0 | 0 |
| **Duration of night sleep** |  |  |  |  |
| ≤ 5 h | 112 | 1.76 | 1.0 (-0.1; 2.0) | 0.8 (-0.3; 1.8) |
| 5.1 – 6.9 h | 377 | 1.29 | 0.4 (-0.2; 1.1) | 0.4 (-0.2; 1.1) |
| ≥ 8 h | 240 | 1.39 | 0.5 (-0.3; 1.3) | 0.5 (-0.3; 1.3) |
| 7 – 7.9 h (ref) | 516 | 0.94 | 0 | 0 |
| **Total sleep** |  |  |  |  |
| ≤ 5 h | 72 | 2.05 | 1.3 (0.0; 2.5) | 1.1 (-0.2; 2.4) |
| 5.1 – 6.9 h | 397 | 1.27 | 0.4 (-0.2; 1.1) | 0.4 (-0.3; 1.1) |
| ≥ 8 h | 250 | 1.42 | 0.6 (-0.2; 1.3) | 0.6 (-0.2; 1.3) |
| 7 – 7.9 h (ref) | 523 | 0.94 | 0 | 0 |
| **Daytime napping** |  |  |  |  |
| Regular | 98 | 0.93 | -0.2 (-1.3; 0.8) | -0.1 (-1.2; 1.0) |
| No / irregular (ref) | 1,146 | 1.23 | 0 | 0 |
| **Any regular sleep disturbance** |  |  |  |  |
| Yes | 367 | 1.31 | 0.3 (-0.3; 0.9) | 0.3 (-0.4; 0.9) |
| no (ref) | 878 | 1.16 | 0 | 0 |

T0: time of baseline visit; T1: time of second visit

Model 1: adjusted for age, sex and weight at baseline

Model 2: adjusted for age, sex, weight at baseline, alcohol intake, smoking, accordance with dietary guidelines, metabolic equivalents / week, education, marital stage, subjective health, stress. For daytime napping as the exposure variable, additional adjustment for sleep duration; for sleep duration as the exposure variable, additional adjustment for any regular sleep disturbances.

**Supplementary table 4b.**

Linear regression models analyzing associations between sleep characteristics at T0 and weight change between T0 and T1 (regression coefficients with 95% confidence intervals): The Heinz Nixdorf Recall study.

Only subjects working less than 15 hours / week at baseline (N=2,172)

|  | **N** | **Mean weight change (kg)** | **Model 1**  **ß (95% CI) (kg)** | **Model 2**  **ß (95% CI) (kg)** |
| --- | --- | --- | --- | --- |
| **Duration of night sleep** |  |  |  |  |
| < 6 h | 261 | 0.33 | 0.0 (-0.6; 0.7) | 0.0 (-0.7; 0.7) |
| > 8 h | 202 | 0.86 | 0.8 (0.1; 1.5) | 0.7 (0.0; 1.5) |
| 6 – 8 h (ref) | 1709 | 0.23 | 0 | 0 |
| **Duration of night sleep** |  |  |  |  |
| ≤ 5 h | 232 | 0.35 | 0.1 (-0.6; 0.9) | 0.1 (-0.7; 0.8) |
| 5.1 – 6.9 h | 487 | 0.47 | 0.4 (-0.2; 1.0) | 0.3 (-0.2; 0.9) |
| ≥ 8 h | 800 | 0.33 | 0.3 (-0.2; 0.8) | 0.2 (-0.3; 0.7) |
| 7 – 7.9 h (ref) | 653 | 0.11 | 0 | 0 |
| **Total sleep** |  |  |  |  |
| ≤ 5 h | 155 | 0.57 | 0.3 (-0.6; 1.2) | 0.3 (-0.6; 1.1) |
| 5.1 – 6.9 h | 495 | 0.43 | 0.3 (-0.2; 0.9) | 0.3 (-0.3; 0.9) |
| ≥ 8 h | 868 | 0.29 | 0.2 (-0.3; 0.7) | 0.2 (-0.3; 0.7) |
| 7 – 7.9 h (ref) | 653 | 0.15 | 0 | 0 |
| **Daytime napping** |  |  |  |  |
| Regular | 441 | -0.17 | -0.3 (-0.8; 0.2) | -0.3 (-0.8; 0.3) |
| No / irregular (ref) | 1,731 | 0.42 | 0 | 0 |
| **Any regular sleep disturbance** |  |  |  |  |
| yes | 1,036 | 0.09 | -0.2 (-0.6; 0.2) | -0.3 (-0.7; 0.2) |
| no (ref) | 1,136 | 0.49 | 0 | 0 |

T0: time of baseline visit; T1: time of second visit

Model 1: adjusted for age, sex and weight at baseline

Model 2: adjusted for age, sex, weight at baseline, alcohol intake, smoking, accordance with dietary guidelines, metabolic equivalents / week, education, marital stage, subjective health, stress. For daytime napping as the exposure variable, additional adjustment for sleep duration; for sleep duration as the exposure variable, additional adjustment for any regular sleep disturbances.
