# Supplementary material for: Associations between sleep characteristics and weight gain in an older population: results of the Heinz Nixdorf Recall Study
Source: Nutr Diabetes. 2016 Aug 15;6(8):e225–. doi: 10.1038/nutd.2016.32 (PMC5022146; doi:10.1038/nutd.2016.32)
Supplement: Supplementary Table 5 [file nutd201632x5.docx]

**Supplementary tables 5a and 5b**

Linear regression models analyzing associations between sleep characteristics at T0 and weight change between T0 and T1 (regression coefficients with 95% confidence intervals): The Heinz Nixdorf Recall study (N=3,751)

Stratified analyses by weight change ≥ / < 5 %.

**Weight change ≥ 5% (N=775):**

|  | **N** | **Mean weight change (kg)** | **Model 1**  **ß (95% CI) (kg)** | **Model 2**  **ß (95% CI) (kg)** |
| --- | --- | --- | --- | --- |
| **Duration of night sleep** |  |  |  |  |
| < 6 h | 109 | 7.61 | 0.2 (-0.4; 0.9) | 0.2 (-0.5; 0.9) |
| > 8 h | 53 | 7.61 | 0.6 (-0.4; 1.5) | 0.6 (-0.4; 1.5) |
| 6 – 8 h (ref) | 613 | 7.05 | 0 | 0 |
| **Duration of night sleep** |  |  |  |  |
| ≤ 5 h | 96 | 7.79 | 0.5 (-0.2; 1.3) | 0.4 (-0.4; 1.2) |
| 5.1 – 6.9 h | 227 | 7.14 | 0.1 (-0.5;0.7) | 0.0 (-0.6; 0.6) |
| ≥ 8 h | 212 | 7.12 | 0.2 (-0.5; 0.8) | 0.1 (-0.5; 0.8) |
| 7 – 7.9 h (ref) | 240 | 6.99 | 0 | 0 |
| **Any regular sleep disturbance** |  |  |  |  |
| yes | 299 | 7.11 | -0.1 (-0.6; 0.4) | -0.1 (-0.6; 0.4) |
| no (ref) | 476 | 7.21 | 0 | 0 |

**Weight change < 5% (N=2,976):**

|  | **N** | **Mean weight change (kg)** | **Model 1**  **ß (95% CI) (kg)** | **Model 2**  **ß (95% CI) (kg)** |
| --- | --- | --- | --- | --- |
| **Duration of night sleep** |  |  |  |  |
| < 6 h | 348 | -1.08 | 0.0 (-0.5; 0.4) | 0.1 (-0.4; 0.5) |
| > 8 h | 191 | -0.89 | 0.2 (-0.3; 0.8) | 0.3 (-0.3; 0.9) |
| 6 – 8 h (ref) | 2437 | -1.01 | 0 | 0 |
| **Duration of night sleep** |  |  |  |  |
| ≤ 5 h | 306 | -1.11 | 0.0 (-0.5; 0.5) | 0.1 (-0.4; 0.7) |
| 5.1 – 6.9 h | 745 | -1.03 | 0.1 (-0.3; 0.4) | 0.1 (-0.3; 0.5) |
| ≥ 8 h | 886 | -0.95 | 0.2 (-0.2; 0.5) | 0.2 (-0.2; 0.5) |
| 7 – 7.9 h (ref) | 1,039 | -1.02 | 0 | 0 |
| **Any regular sleep disturbance** |  |  |  |  |
| yes | 1,198 | -1.21 | -0.1 (-0.4; 0.1) | -0.1 (-0.4; 0.2) |
| no (ref) | 1,778 | -0.88 | 0 | 0 |

T0: time of baseline visit; T1: time of second visit

Model 1: adjusted for age, sex and weight at baseline

Model 2: adjusted for age, sex, weight at baseline, alcohol intake, smoking, accordance with dietary guidelines, metabolic equivalents / week, education, marital stage, subjective health, stress. For sleep duration as the exposure variable, additional adjustment for any regular sleep disturbances.
