# Supplementary material for: Associations between sleep characteristics and weight gain in an older population: results of the Heinz Nixdorf Recall Study
Source: Nutr Diabetes. 2016 Aug 15;6(8):e225–. doi: 10.1038/nutd.2016.32 (PMC5022146; doi:10.1038/nutd.2016.32)
Supplement: Supplementary Information [file nutd201632x6.docx]

**Stress scale used to assess stress levels**

1. Does it normally cost you a lot of time and energy do to the housework (e.g., childcare, cooking and cleaning)?

No

Yes, and this bothers me (not at all / a little / much / very much)

1. Do you normally receive appropriate acknowledgement or reward for your housework (e.g., childcare, cooking and cleaning)?

Yes

No, and this bothers me (not at all / a little / much / very much)

1. Do you often have to behave considerately to keep up a good relationship with your life partner?

I have no life partner. (Go to question 7)

No

Yes, and this bothers me (not at all / a little / much / very much)

1. Is there always a feeling of mutual confidence between you and your partner?

Yes

No, and this bothers me (not at all / a little / much / very much)

1. Is there always a balance between what you give your partner and what you receive from him?

Yes

No, and this bothers me (not at all / a little / much / very much)

1. Is your partnership always characterized by mutual understanding?

Yes

No, and this bothers me (not at all / a little / much / very much)

1. Do you often give special help to your family (e.g., caring for parents / parents-in-law) without being rewarded appropriately?

No

Yes, and this bothers me (not at all / a little / much / very much)

1. Do you often have to be considerate for the well-being of your children?

I have no children. (Go to question 11.)

No

Yes, and this bothers me (not at all / a little / much / very much)

1. Do you normally feel appreciated by your children?

Yes

No, and this bothers me (not at all / a little / much / very much)

1. Is there an important aim in the education of your children you have not yet achieved?

No

Yes, and this bothers me (not at all / a little / much / very much)

1. Do you feel much deceived or hurt by a person in whom you had much confidence?

No

Yes, and this bothers me (not at all / a little / much / very much)

1. Has anybody wronged or deceived you, and you did not get compensation afterwards?

No

Yes, and this bothers me (not at all / a little / much / very much)

1. Do you have financial problems?

No

Yes, and this bothers me (not at all / a little / much / very much)

For each item, study subjects were assigned

- one point, if they stated that they did not have the respective problem,
- two points, if they had the problem, and it bothered them “not at all”,
- three points, if they had the problem, and it bothered them “ a little”,
- four points, if they had the problem, and it bothered them “much”,
- five points, if they had the problem, and it bothered them “very much”.

Subjects were assigned one point for each of the items 3 to 6 if they had no partner, and one point for each of the items 8 to 10 if they had no children.

If the number of missing values exceeded three, the stress score was not calculated. If the number of missing values did not exceed three, missing values were imputed by the arithmetic mean of the available values, and the total stress score was calculated as the mean value of all 13 items.
